# Realistic Image Rendition Using a Variable Exponent Functional Model for Retinex

**DOI:** 10.3390/s16060832

**Published:** 2016-06-07

**Authors:** Zeyang Dou, Kun Gao, Bin Zhang, Xinyan Yu, Lu Han, Zhenyu Zhu

**Affiliations:** 1 Key Laboratory of Photoelectronic Imaging Technology and System, Ministry of Education of China, Beijing Institute of Technology, Beijing 100081, China; douzeyang123@163.com (Z.D.); hanlubest1@163.com (L.H.); zhenyu0212@sina.cn (Z.Z.); 2 School of Science, Communication University of China, Beijing 100024, China; zhangbin628@cuc.edu.com (B.Z.); yuxinyan@cuc.edu.com (X.Y.)

**Keywords:** Retinex, variable exponent functional, illumination removal, halo artifact, image rendition

## Abstract

The goal of realistic image rendition is to recover the acquired image under imperfect illuminant conditions, where non–uniform illumination may degrade image quality with high contrast and low SNR. In this paper, the assumption regarding illumination is modified and a variable exponent functional model for Retinex is proposed to remove non–uniform illumination and reduce halo artifacts. The theoretical derivation is provided and experimental results are presented to illustrate the effectiveness of the proposed model.

## 1. Introduction

**Figure 1:**
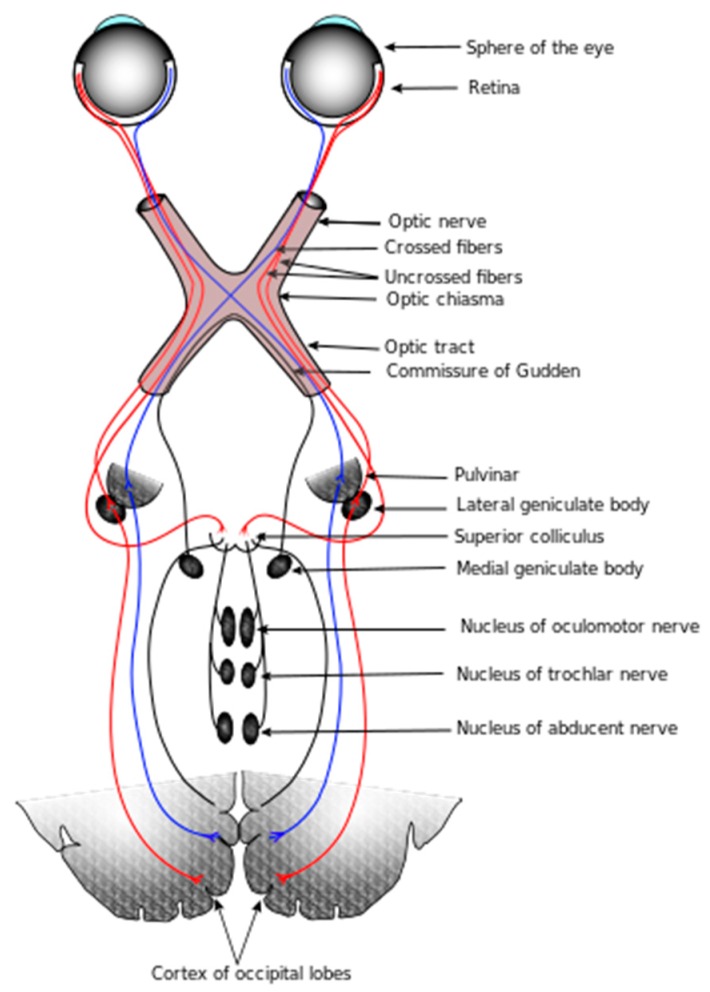
Elementary structure of the retina, lateral geniculate nucleus, and cortex.

**Figure 2:**
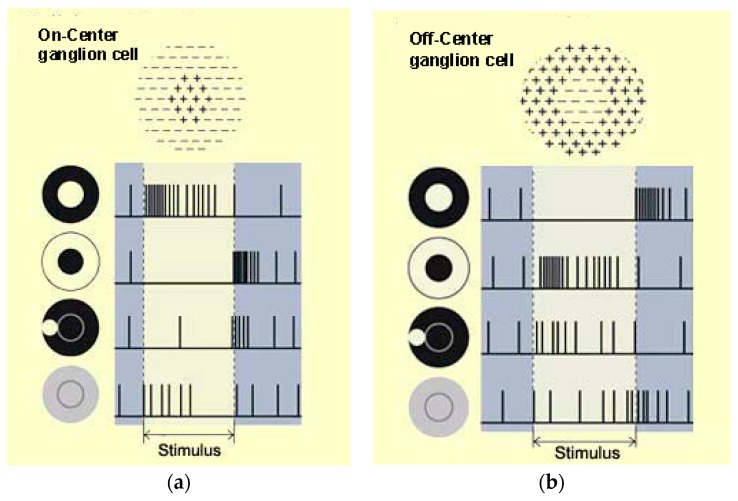
Receptive field in the retina. (**a**) on-center ganglion cell; (**b**) off-center ganglion cell.

Realistic image rendition aims to represent human perception of natural scenes. The meaning of “realistic” is to provide machine vision with ideal images according to the human visual system. A complete visual pathway includes the optic nerve, retina, optic tract, optic chiasm, superior colliculus, lateral geniculate nucleus, optic radiation, and cortex, as shown diagrammatically in Figure 1 [1]. The main features of realistic image rendition include color constancy, image enhancement, high dynamic range compression, *etc*. The physiological basis for color constancy involves specialized neurons in the primary visual cortex that compute local ratios of cone activity [2], which is the same calculation as Land’s Retinex algorithm [3,4] used to achieve color constancy. The existence of these specialized cells, double–opponent cells, has been proven using receptive field mapping. Receptive field [5,6] is the basic unit of visual information processing, and can be separated into two types: On–Center and Off–Center ganglion cells. Figure 2 shows the receptive field in the retina.

Algorithms of realistic image rendition based on visual characteristics generally include Retinex algorithms for color constancy. The word “Retinex” is a combination of “retina” and “cortex”. The aim of Retinex theory is to tell whether human eyes can determine reflectance when both the illumination and reflectance are unknown. Land and McCann [6] first proposed the Retinex theory, a path-based algorithm, as a model of color perception of the human visual system (HSV). Many algorithms [7,8,9] are based on this approach, which differ in how the path is selected. However, these methods have high computation complexity and require numerous parameters. McCann [10,11,12] replaced the path calculation by a recursive matrix computation which greatly improved computational efficiency. However, the terminal criterion is not clear and can strongly influence the result. In PDE based models [13], the Retinex principles are often translated into a physical form. These algorithms are developed based on solving a Poisson equation which can yield fast and exact implementation using only two fast Fourier transforms. The main assumption in this algorithm type is that the reflectance performs as the sharp details in the image, while illumination varies smoothly. Based on the assumptions used in PDE formulations, Kimmel *et al.* [14] proposed a general variational model for the Retinex problem that unified previous methods. Ma and Osher [15,16] proposed a total variation and nonlocal total variation(TV) regularized model using the same assumptions. Ng *el at.* [17] investigated the TV model with more constraints. Recently, Liang and Zhang [18] established a new higher order total variation L1 decomposition model (HoTVL1) which can correct the piecewise linear shadows. Zosso [19,20] proposed a unifying Retinex model based on non-local differential operators.

To the best of our knowledge, almost all of the important assumptions about illumination in existing Retinex models require spatial smoothness. However, many images with non-uniform illuminations have non smooth illumination, actually. In this paper, we assume:

(a) The reflecting object a Lambertian reflector and reflectance corresponds to sharp details in the image;
(b) Illumination is smooth in most regions, but may contain non-smooth part(s).

Based on these assumptions, we propose a new Retinex model using a variable exponent functional. We assume that the illumination function belongs to some Sobolev space with variable exponents. The proposed model solution existence is proved here. Although the proposed model is developed for specific cases, it can also be applied to general degraded images and significantly reduces the halo artifact.

In Section 2, we argue the reasonability of the assumption and present the proposed model. We also present a proof of solution existence for the proposed model, and introduce an efficient iterative solution method. In Section 3, we present several numerical examples to demonstrate the effectiveness of the proposed model. Concluding remarks are presented in Section 4.

## 2. New Assumption and Proposed Model

### 2.1. New Assumption

**Figure 3:**
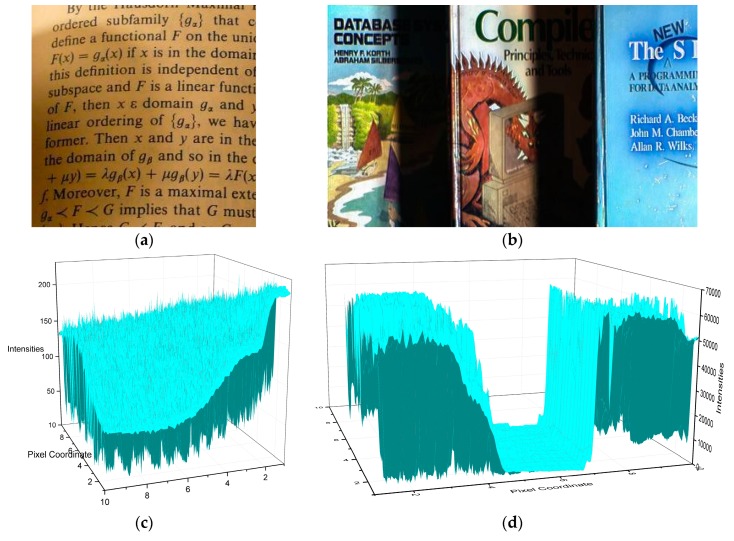
Images with different illumination and the corresponding surfaces: (**a**) text image; (**b**) book image; (**c**) surface of (**a**); (**d**) surface of (**b**).

**Figure 4:**
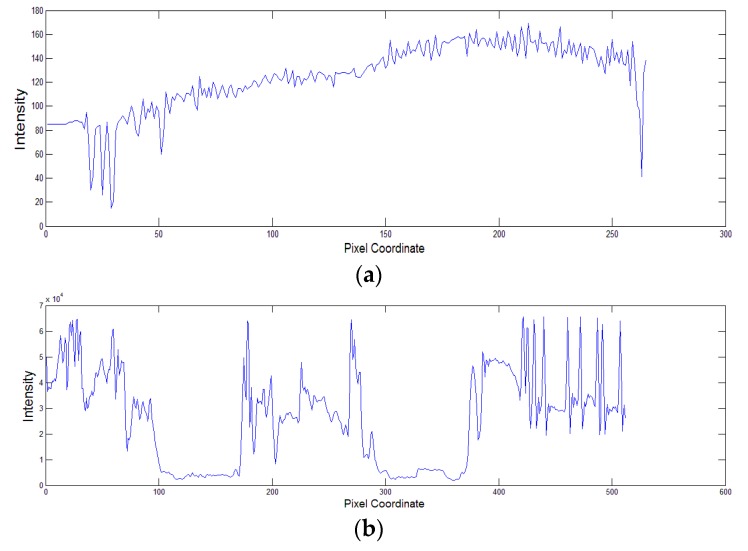
A single row extracted from: (**a**) text image; (**b**) book image.

To illustrate the proposed assumption, let us consider the images with different illumination conditions and their corresponding surfaces in Figure 3. The corresponding surfaces illustrate the shadow shapes. The illumination of the text image varies smoothly, whereas that of the book image has an apparent non-smooth component. Figure 4 shows a single row from the two images, illustrating the text image curve changes relatively smooth in the shadow area, while that of the book image changes dramatically at the edge of the shadow and relatively smooth in the interior of the shadow.

The above examples support the proposed assumption. Indeed, every severe non-uniform illumination case is likely to have a non-smooth part. Our aim is to extract illumination images and recover realistic images.

### 2.2. Proposed Model

First, we introduce the variable exponent functional and some related models. Blomgren *et al.* [21] proposed the variable exponent functional for image denoising problems. They tried to minimize:

(1) $$E(u)=\int_{\Omega }|\nabla u{|}^{p(|\nabla u|)}dx$$

where *u* is the image function and *p* is a monotonically decreasing function with $\lim_{s\to 0}p(s)=2$, lim*_s_*_→∞_*p*(*s*) = 1. Choosing *p* = 1 produces the widely used Rudin-Osher-Fatemi (ROF) model [22] which preserves edge sharpness, but often causes the “staircase” effect; *p* = 2 produces isotropic diffusion, which avoids the “staircase” effect but smears edges. Thus, it is natural to combine their benefits with a variable exponent. However, because *p* relies on ∇u, it is difficult to establish the lower semi continuity property of the functional. Chen *et al.* [23] proposed a variable exponent linear growth functional model for image denoising, enhancement and restoration, which is extended by Li *et al.* [24], using variable exponent functionals in image restoration.

For simplicity, we formulate and discuss our model based on grayscale images. For color images, we simply map the color into HSV(hue, saturation, value) color space, process only the V channel, then transform it back to the RGB domain. This method is called HSV Retinex [14,17].

**Figure 5:**
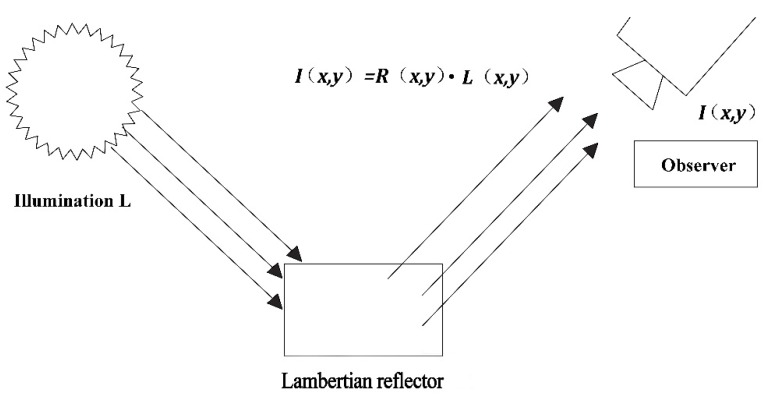
Schematicdiagram for Retinex.

Let *I* be an image defined in image domain Ω. The primary goal of Retinex theory is to decompose *I* into the reflectance image, R, and the illumination image, *L*, as shown in Figure 5, such that, at each point in the image domain [25]:

(2) I=R⋅L

and following [14,17], we may further assume that:

L≥I>0

We first convert Equation (2) into the logarithmic domain:

i=log(I), l=log(L), r=log(R)

so that:

i=l+r

Based on our new assumption, the illumination image may contain non-smooth parts. Weuse a total variation like regularizer near non-smooth parts and a Tikhonov like regularizer for smooth parts. We minimize the objective function as follows:

(3) $$E(l)=\int_{\Omega }|\nabla l-\nabla i{|}^{2}dx+\lambda \int_{\Omega }|\nabla l{|}^{p(x)}dx$$

where λ is a positive number, and $p(x)=1+\frac{1}{1+w|\nabla d{|}^{2}}$, where *d* is the ideal illumination image, discussed in Section 2.4.2. The first fidelity term on the right side of model (3) measures the similarity of the gradient between the illumination and the original image, and the second is the regularization term. Clearly, p→1 near the edges of *d* where the gradient is large, and so the regularizer is similar to a TV regularizer which can preserve edges; p→2 in the homogeneous regions where the gradient is small, and here the regularizer is similar to a Tikhonov like regularizer, which is superior to total variation. In other regions, the penalty is adjusted by *p*(*x*).

The classical Retinex algorithm uses a Gaussian filter, equivalent to a Tikhonov regularizer, to obtain the illumination image. However, a Gaussian filter smears edges, which is the main cause of halo artifacts [26]. Using the adaptive TV like regularizer for the high contrast edges in the image, our model not only prevents halo artifacts but also extracts the edges of non-uniform illumination from the image.

### 2.3. Solution Existence

Let us recall some definitions and basic properties of variable exponent Lebesgue and Sobolev spaces, following [24,27].

*Definition*  (variable exponent spaces): Let Ω be a bounded open set with Lipchitz boundary and p(x):Ω→[1,+∞) be a measurable function, with the family of all measurable functions on Ω being P(Ω). We define a functional, which is also called modular:

$$Q_{p(x)}(u)=\int_{\Omega }|u{|}^{p(x)}dx$$

and a norm:

$$\left||u{\left|\right|}_{p(x)}=\inf\{\lambda >0:Q_{p(x)}(u/\lambda )\leq 1\right\}$$

Then the variable exponent Lebesgue and Sobolev spaces are, respectively:

$$L^{p(x)}(\Omega )=\{u:\Omega \to R|\;||u|{|}_{p(x)}<\infty \}$$

and:

$$W^{1,p(x)}(\Omega )=\{u:\Omega \to R|\;u\in L^{p(x)}(\Omega ),\nabla u\in L^{p(x)}(\Omega )\}$$

With the norm $||u|{|}_{1,p(x)}=||u{\left|\right|}_{p(x)}+||\nabla u{\left|\right|}_{p(x)}$, $W^{1,p(x)}(\Omega )$ becomes a Banach space.

**Lemma 1.**  *(relationship between modular and norm [27]): Let* $Q_{p(x)}$ *be a modular on* X *and* u∈X*, then* $\left||u{\left|\right|}_{p(x)}\leq Q_{p(x)}(u\right)$*+1*.

**Lemma 2.**  *(embedding theorem [24]): Let* p(x),q(x)∈P(Ω) *, and* p(x)≤q(x) *for a.e.* x∈Ω*. Then* $L^{q(x)}(\Omega )$ *is continuously embedded in* $L^{p(x)}(\Omega )$.

**Lemma 3.**  *(convexity [27]): Let* $F(\nabla l,x)=|\nabla l{|}^{p(x)}$ *, with* $p(x)=1+\frac{1}{1+w|\nabla d{|}^{2}}$ *as in model (3). Then for each* x*,* F(ξ,x) *is convex in* ξ.

**Lemma 4.**  *(weak lower semi continuity [27]) Let* F(ξ,x) *be bounded from below, and the map* ξ→F(ξ,x) *is convex in each* x∈Ω*. Then the energy functional,* $I=\int_{\Omega }F(\nabla l,x)dx$*, is weak lower semi-continuous in* $W^{1,p(x)}$.

**Theorem 1.**  *Let* $\Omega \subset R^{2}$ *be a bounded open set with Lipchitz boundary,* $i\in W^{1,p(x)}(\Omega )\cap L^{2}(\Omega )$*, then the minimization problem*:

$$\underset{l\in W^{1,p(x)}(\Omega )\cap L^{2}(\Omega )}{\min}\{E(l)=\int_{\Omega }|\nabla l-\nabla i{|}^{2}dx+\lambda \int_{\Omega }|\nabla l{|}^{p(x)}dx\}$$

has a minimizer $l\in W^{1,p(x)}(\Omega )\cap L^{2}(\Omega )$.

**Proof of Theorem 1.**  Let ${\left\{l_{k}\right\}}_{k=1}^{\infty }$, $l_{k}\in W^{1,p(x)}(\Omega )\cap L^{2}(\Omega )$ be the minimizing sequence for E(l). Then:

$$\int_{\Omega }|\nabla l_{k}-\nabla i{|}^{2}dx<M\text{ and }\int_{\Omega }|\nabla l_{k}{|}^{p(x)}dx<M$$

where *M* denotes a universal positive constant that may differ from line to line. Hence $\int_{\Omega }|\nabla l_{k}{|}^{2}dx<M$. Thanks to Poincare inequality, we have $\int_{\Omega }l_{k}^{2}dx<M$, and from Lemma 2, $L^{2}(\Omega )\subset L^{p(x)}(\Omega )$. Therefore, $\int_{\Omega }|l_{k}{|}^{p(x)}dx<M$, and together with the inequality $\int_{\Omega }|\nabla l_{k}{|}^{p(x)}dx<M$, we obtain $Q_{p(x)}(l_{k})+Q_{p(x)}(\nabla l_{k})<M$. This implies that ${\left\{l_{k}\right\}}_{k=1}^{\infty }$ is a uniformly bounded sequence in $W^{1,p(x)}(\Omega )$ due to Lemma 1, and ${\left\{l_{k}\right\}}_{k=1}^{\infty }$ is also uniformly bounded in $L^{2}(\Omega )$. Since $W^{1,p(x)}(\Omega )\cap L^{2}(\Omega )$ is a reflexive Banach space, up to a subsequence, there exists $l^{*}\in W^{1,p(x)}(\Omega )\cap L^{2}(\Omega )$ such that $\left\{l_{k_{j}}\right\}$ converges weakly to $l^{*}$ in $W^{1,p(x)}(\Omega )\cap L^{2}(\Omega )$. From Lemma 4, E(l) is lower semi continuous in $W^{1,p(x)}(\Omega )\cap L^{2}(\Omega )$. Thus:

$$E(l^{*})\leq \underset{k\to \infty }{\lim}E(l_{k})=\underset{l\in W^{1,p(x)}(\Omega )\cap L^{2}(\Omega )}{\inf}E(l)$$

Therefore, $l^{*}$ is the minimum point of E(l). ☐

### 2.4. Implementation

We formulate the basic procedure for solving problem Equation (3) following the split Bregman [28,29,30,31,32,33] technique. We solve the minimization by introducing an auxiliary variable *b*:

(4) $$\min\{\int_{\Omega }|\nabla l-\nabla i{|}_{}^{2}dx+\lambda \int_{\Omega }|b{|}^{p(x)}dx\}\text{ subject to }b=\nabla l$$

By adding one quadratic penalty function term, we convert Equation (4) to an unconstrained splitting formulation:

(5) $$\min\{\int_{\Omega }|\nabla l-\nabla i{|}^{2}dx+\lambda {\int_{\Omega }|b{|}^{p(x)}dx+\gamma \int_{\Omega }\left|b-\nabla l\right|}^{2}dx\}$$

where γ is a positive parameter which controls the weight of the penalty term. Similar to the split Bregman iteration, we propose the scheme:

(6) $$\{\begin{array}{c} (l^{k+1},b^{k+1})=\underset{}{\underset{l,b}{\arg\min}\int_{\Omega }|\nabla l-\nabla i{|}^{2}dx+\lambda \int_{\Omega }|b{|}^{p(x)}dx+\gamma \int_{\Omega }|b-\nabla l-t^{k}{|}^{2}dx}\\ t^{k+1}=t^{k}+\nabla l^{k+1}-b^{k+1} \end{array}$$

Alternatively, this joint minimization problem can be solved by decomposing into several subproblems.

#### 2.4.1. Subproblem *l* with Fixed *b* and *t*

Given the fixed variable *b^k^* and *t^k^* , our aim is to find the solution of the problem:

(7) $$l^{k+1}=\underset{l}{\arg\min}\int_{\Omega }|\nabla l-\nabla i{|}^{2}dx+\gamma \int_{\Omega }|b^{k}-\nabla l-t^{k}{|}^{2}dx$$

which has the optimality condition:

(8) $$(\gamma +1)\Delta l=\gamma \nabla \cdot (b^{k}-t^{k})+\Delta i$$

where $b=(b_{x},b_{y})$ and $t=(t_{x},t_{y})$. Since the discrete system is strictly diagonally dominant with Neumann boundary condition, the most natural choice is the Gauss-Seidel method. The Gauss-Seidel solution to this subproblem can be written componentwise as:

$$\begin{array}{l} l_{i,j}^{k+1}=\frac{\gamma }{4(\gamma +1)}(b_{x,i-1,j}^{k}+b_{y,i,j-1}^{k}-b_{x,i,j}^{k}-b_{y,i,j}^{k}+t_{x,i-1,j}^{k}+t_{y,i,j-1}^{k}-t_{x,i,j}^{k}-t_{y,i,j}^{k})+\\ \frac{1}{4(\gamma +1)}(4i_{i,j}^{k}-i_{i+1,j}^{k}-i_{i-1,j}^{k}-i_{i,j-1}^{k}-i_{i,j+1}^{k})+\frac{\gamma +1}{4(\gamma +1)}(l_{i+1,j}^{k}+l_{i-1,j}^{k}+l_{i,j+1}^{k}+l_{i,j-1}^{k}) \end{array}$$

Note that this subproblem can also be solved by FFT with periodic boundary condition.

#### 2.4.2. Subproblem *b* with Fixed *l* and *t*

Similarly, we solve:

(9) $$b^{k+1}=\underset{b}{\arg\min}\lambda \int_{\Omega }|b{|}^{p(x)}dx+\gamma \int_{\Omega }|b-\nabla l^{k+1}-t^{k}{|}^{2}dx$$

which has the optimality condition:

(10) $$\{\begin{array}{c} \lambda p(x)|b{|}^{p(x)-2}b_{x}+2\gamma (b_{x}-\nabla_{x}l_{}^{k+1}-t_{x}^{k})=0\\ \lambda p(x)|b{|}^{p(x)-2}b_{y}+2\gamma (b_{y}-\nabla_{y}l_{}^{k+1}-t_{y}^{k})=0 \end{array}$$

where $\nabla l=(\nabla_{x}l,\nabla_{y}l)$. If $b_{x}$ and $b_{y}$ are not zero, then:

(11) $$b_{x}=\frac{\nabla_{x}l_{}^{k+1}+t_{x}^{k}}{\nabla_{y}l_{}^{k+1}+t_{y}^{k}}b_{y}$$

Substituting Equation (11) into Equation (10):

(12) $$\mathrm{sgn}(b_{y})Tb_{y}^{p(x)-1}+2\gamma (b_{y}-\nabla_{y}l_{}^{k+1}-t_{y}^{k})=0$$

where $T=\lambda p(x){\left({\left(\frac{\nabla_{x}l_{}^{k+1}+t_{x}^{k}}{\nabla_{y}l_{}^{k+1}+t_{y}^{k}}\right)}^{2}+1\right)}^{\frac{p(x)-2}{2}}$. Note that:

(13) $$\mathrm{sgn}(b_{x})=\mathrm{sgn}(\nabla_{x}l_{}^{k+1}+t_{x}^{k})$$

(14) $$\mathrm{sgn}(b_{y})=\mathrm{sgn}(\nabla_{y}l_{}^{k+1}+t_{y}^{k})$$

So Equation (12) can be expressed as:

(15) $$\mathrm{sgn}(\nabla_{y}l_{}^{k+1}+t_{y}^{k})Tb_{y}^{p(x)-1}+2\gamma (b_{y}-\nabla_{y}l_{}^{k+1}-t_{y}^{k})=0$$

Unfortunately, we cannot obtain the explicit solution of the Equation (15). We can use the Newton method to get an approximate solution. If $b_{y}^{}$ is solved, $b_{x}^{}$ can be easily determined using Equations (11) and (13). The process is shown as Algorithm 1.

**Algorithm 1:** (Newton’s method)Input: $b_{y}^{0}=\nabla_{y}l_{}^{k+1}$ If $\nabla_{y}l_{}^{k+1}+t_{y}^{k}=0$  Output $b_{y}^{k+1}=0$ Else  while not converged $$b_{y}^{j+1}=\text{sign}(\nabla_{y}l_{}^{k+1}+t_{y}^{k})\text{max}\{b_{y}^{j}-\frac{T{\left(b_{x}^{j}\right)}^{p(x)-1}+2\gamma (b_{y}^{j}-|\nabla_{y}l_{}^{k+1}+t_{y}^{k}|)}{(p(x)-1)T{\left(b_{x}^{j}\right)}^{p(x)-2}+2\gamma b_{y}^{j}},0\}$$  End Output $b_{y}^{k+1}$ If $\nabla_{x}l_{}^{k+1}+t_{x}^{k}=0$  Output $b_{x}^{k+1}=0$ Else $b_{x}^{k+1}=\frac{\nabla_{x}l_{}^{k+1}+t_{x}^{k}}{\nabla_{y}l_{}^{k+1}+t_{y}^{k}}b_{y}^{k+1}$ End End

Another problem is that in practice we don't know *d* in *p*(*x*). We have tested two ways to approximate *d*. One way is to use edge preserving filter (e.g., bilateral filter) to give an approximation of *d* and keep the exponent fixed during the iteration; Another way is to replace *d* with $G(l^{k+1})$ during the iteration and G(⋅) represents the Gauss convolution operator, In most cases, both methods can generate similar prominent results. However, in some cases, dynamic approximation would give better results than fixed approximation because dynamic approximation can give a more accurate approximation of *d* along with the iteration. To illustrate this, consider the associated heat flow to problem Equation (3):

(16) $$\begin{array}{ll} l_{t} & =2(\Delta l+\Delta i)+\lambda p(x)\nabla \cdot (|\nabla l{|}^{p(x)-2}\nabla l)\\ & =(2+\lambda p(x)|\nabla l{|}^{p(x)-2})l_{TT}+(2+\lambda p(x)(p(x)-1)|\nabla l{|}^{p(x)-2})l_{NN} \end{array}$$

where $l_{TT}$ and $l_{NN}$ are the second derivatives of l in the tangent and normal direction to the isophote lines respectively. From Equation (16), we have two critical conclusions:

1. The illumination image, $l^{k+1}$, becomes increasingly smooth over time.
2. Diffusion speed in the tangent direction is always faster than that in the normal direction.

The first conclusion conforms to the smooth assumption of the illumination image. If the illumination image has non smooth parts, then the second conclusion guarantees that the solution can preserve these parts. Thus, $l^{k+1}$ continuously gets closer to *d* with calculation. However, the convergence proof of the algorithm is difficult since the exponent is changing during the iterations. If the exponent is fixed, the convergence proof can be directly obtained because the objective function is fixed and the iteration of split Bregman is monotone decreasing in the function values. The strict proof can be found in references [34,35]. If the exponent changes during the iterations, then the objective function changes as well. The convergence proof in references [34,35] cannot be applied here. However, we have tested numerous experiments and our algorithm did converges in all the tests. We leave it for further work.

#### 2.4.3. Update:

(17) $$t^{k+1}=t^{k}+\nabla l^{k+1}-b^{k+1}$$

#### 2.4.4. Update $l^{k+1}$:

$$l_{}^{k+1}=\max\{l_{}^{k+1},i\}$$

which corresponds to the constraint L≥I>0.

The process is shown as Algorithm 2.

**Algorithm 2:** (Variable Exponent Functional Retinex)Input: Image I Transform into log domain i=log(I+1); Initialization: $l_{}^{0}=i$, $b^{0}=0$, $t^{0}=0$ and k=0 While $\frac{\left||l^{k}-l_{}^{k-1}|\right|}{\left||l_{}^{k}|\right|}\leq \varepsilon$  (1) Given $b^{k}$ and $t^{k}$, update $l_{}^{k+1}$ by solving Equation (8).  (2)Given $l_{}^{k+1}$ and $t_{}^{k}$, update $b_{}^{k+1}$ by using algorithm 1.  (3)Update $t^{k+1}=t^{k}+\nabla l^{k+1}-b^{k+1}$.  (4) $l_{}^{k+1}=\max\{l_{}^{k+1},i\}$;  (5)k=k+1; End Output: Image $R=\frac{\exp(i)}{\exp(l)}$


### 2.5. Relation to Previous Methods

Let us revisit the model in Section 2.2. If we set p(x)=2 and remove the constraint *l* ≥ *i*, our model is equivalent to homomorphic filtering [36]. Retaining the constraint *l* ≥ *i* and fixing *p*(*x*) = 2, it is similar to a random walk, Ng’s model [17] and McCann algorithm [12]. Thus, our proposed model generalizes previous models.

## 3. Numerical Results

We present numerical results to demonstrate the efficiency of the proposed model and algorithm. For color images examples, we use HSV Retinex. We compare our proposed model with three state of the art methods, HoTVL1 [18], Ng’s method [17] and multiscale Retinex [37].

**Figure 6:**
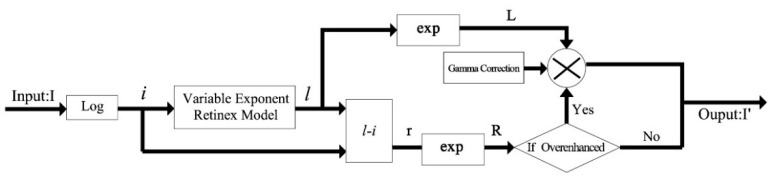
Global framework of the proposed method.

For all the tests, the recovered reflectance of our model is:

(18) $$R=\frac{I}{L}$$

where *L* = exp(*l*) is the illumination function obtained from Section 2.4, and *I* = exp(*i*) is the original image. Note that the reflectance image is sometimes over enhanced, and we add the Gamma correction illumination to the reflectance image after decomposition. The Gamma correction of *L* with parameter *s* is:

(19) $$L\prime =W{\left(\frac{L}{W}\right)}^{\frac{1}{s}}$$

where *W* is the value of the white pixel. Parameter *s* was set to Section 2.2 in the tests. Thus:

(20) I′=L′⋅R

and the global framework of our proposed method is illustrated in Figure 6.

### 3.1. Synthetic Images

**Figure 7:**
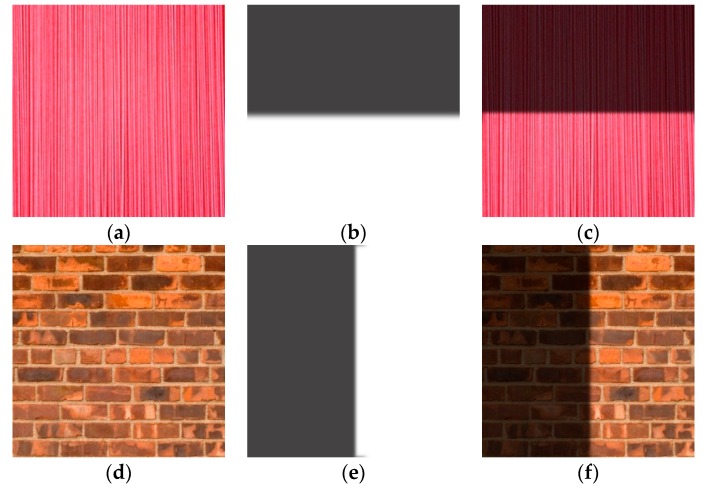
Synthetic example. (**a**,**d**) original image; (**b**,**e**) simulated illumination; (**c**,**f**) synthetic image.

**Figure 8:**
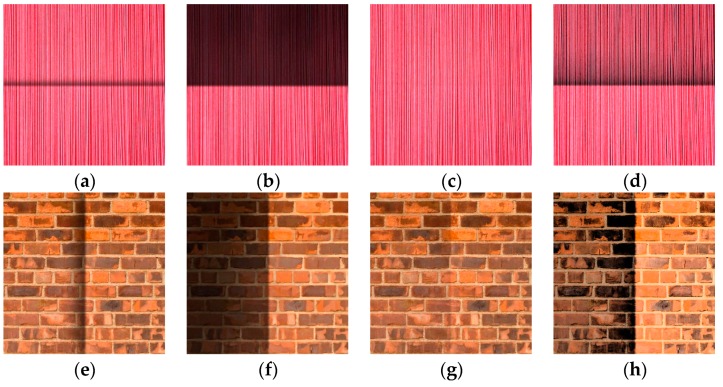
Image reconstruction by different methods. (**a**,**e**) Ng; (**b**,**f**) HoTVL1; (**c**,**g**) proposed; (**d**,**h**) mutiscale Retinex.

**Figure 9:**
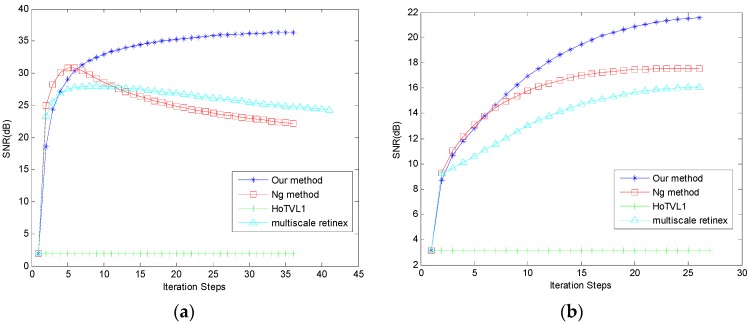
SNR curves. (**a**) For Figure 7a; (**b**) For Figure 7d.

In this subsection, we set λ = 80, γ = 10^3^ and w = 10^9^. Simulated illumination is added to the original texture images, as shown in Figure 7, with the numerical results shown in Figure 8. The recovered image following our proposed method is visually superior. We use signal to noise ratio (SNR) to measure the similarities between the original and recovered images, as shown in Figure 9. SNR from our proposed method is significantly superior to the other methods. We further use structural similarity index (SSIM) and CIEDE2000 color difference to measure the texture similarities and perceptual difference between the original and recovered images respectively, as shown in Table 1 and Table 2. We can see from tables that our proposed method is superior to the other methods. We note that HoTVL1 failed in these tests. The main reason is that the assumption in HoTVL1 is piecewise constant and piecewise linear, which means that the shadow should be piecewise linear. However, this is not the case of these tests. The shadow part is almost a constant and also has sharp edges. Hence the result of HoTVL1 is not satisfactory.

### 3.2. Natural Images

**Figure 10:**
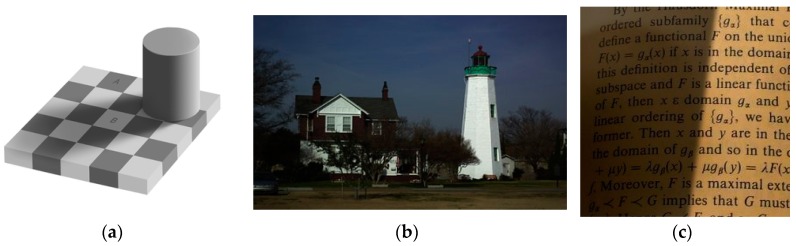
Test images. (**a**) Checkerboard image; (**b**) Tower image; (**c**) Text image.

**Figure 11:**
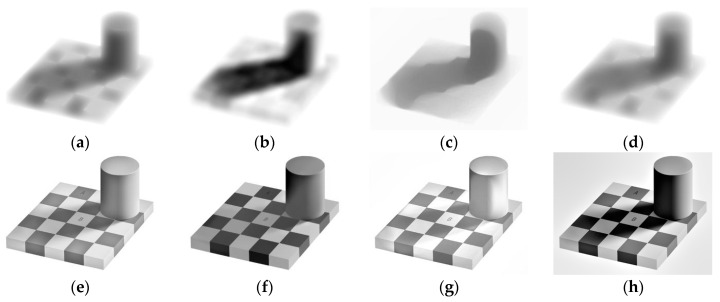
Illumination (**a**–**d**) and reflection (**e**–**h**) images. (**a**,**e**) Ng’s method; (**b**,**f**) HoTVL1; (**c**,**g**) proposed method; (**d**,**h**) multiscale Retinex.

For all tests, we set *λ* = 80, *γ* = 10^3^ and *w* = 10^3^. We begin with Andelson’s checkerboard shadow image, as shown in Figure 10a. Region A looks darker than region B, although they have the same values. Figure 11 shows the reconstructed illumination and reflection images using Ng’s, HoTVL1, multiscale Retinex and our proposed model. HoTVL1 and the proposed method produce superior results to Ng’s method and multiscale Retinex. The recovered illumination using our proposed method contains less reflectance information than HoTVL1, e.g., the outline of the cylinder. Our proposed method also contains less shadow information in the reflectance image than other methods. Table 3 compares the recovered intensity values of the two regions for the four methods. The contrast of the marked areas using our proposed method is superior to the other three methods.

**Figure 12:**
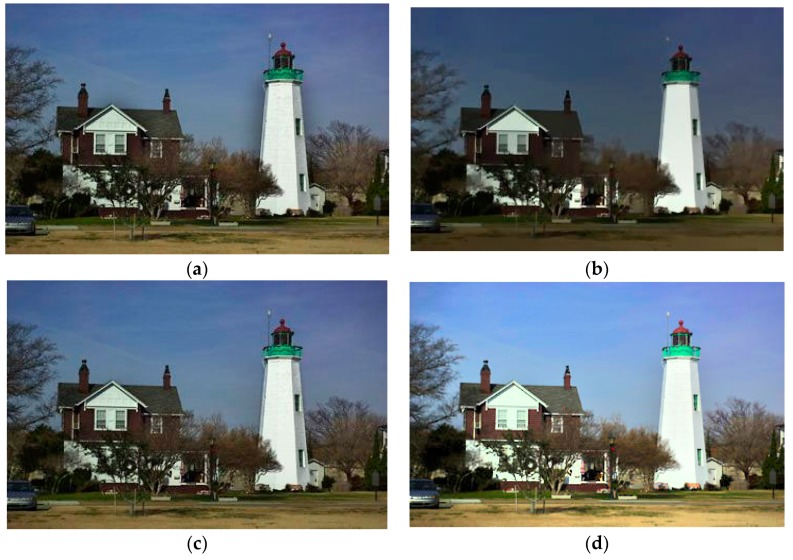
Reproduced degraded image (Figure 10b). (**a**) Ng’s model; (**b**) HoTVL1; (**c**) proposed model; (**d**) multiscale Retinex.

Consider the degraded image shown in Figure 10b. Figure 12 shows the reconstruction for the four methods. Note that in this example, we adopted a Gamma correction step, as discussed above. Our proposed method has superior visual outcome. Ng’s method suffers halo artifacts, e.g., near the edges of the tower and the roof of the building, which rarely appear in HoTVL1, multiscale Retinex and our proposed method. However, many fine structures lost in the HoTVL1 and multiscale Retinex reproduced image, which are retained in our proposed method.

**Figure 13:**
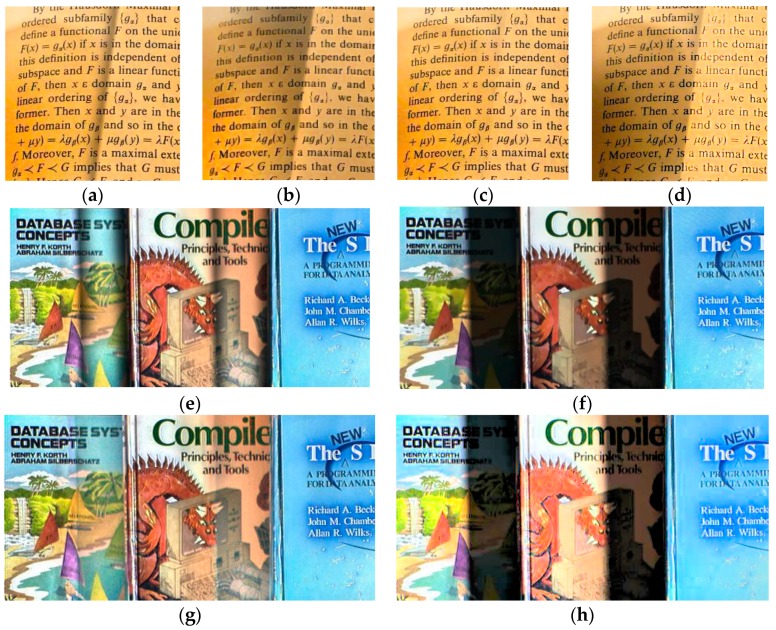
Reproduced shadowed images (Figure 10c and Figure 3b): (**a**,**e**) Ng’s model; (**b**,**f**) HoTVL1; (**c**,**g**) proposed model; (**d**,**h**) multiscale Retinex.

The next illustrative example is recovery of non-uniform degraded images. The two images in Figure 10c and Figure 3b suffer from the strong shadow areas. Figure 13 shows the comparison for the considered methods. The shadow is almost entirely removed by our proposed method, whereas the other methods retain partly shadowed regions.

**Figure 14:**
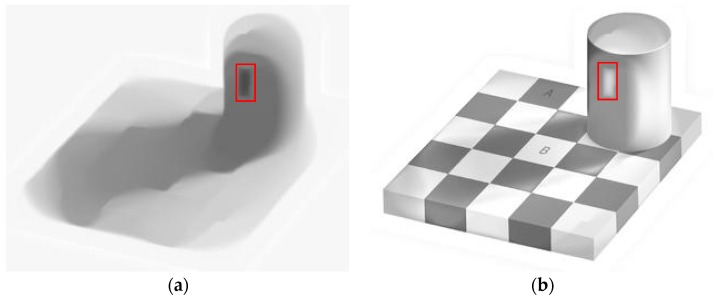
Reproduced illumination and reflectance (Figure 10a) (**a**) Illumination with fixed approximation; (**b**) Reflectance with fixed approximation. Note the circled part by the red square.

In the end, we test the effect of different approximations of *d.* We use bilateral filter to approximate *d* and keep the exponent fixed during the iterations. Figure 14 shows the numerical results. We see in Figure 14 that the illumination and the reflectance of the results are not as good as those in Figure 11. This experiment supports our discussion in Section 2.4.2.

## 4. Conclusions

We proposed a variable exponent functional model for Retinex, proved the existence of the solution for the model and provided the theoretical derivation. The proposed method can be applied to general degraded cases as well. Experimental results validatethat our proposed method can remove non-uniform illumination and significantly reduce halo artifacts.

## Figures and Tables

**Table 1:** SSIM of the four methods.

Image	Ng	HoTVL1	Proposed Method	Multiscale Retinex
Figure 7a	0.9076	0.6342	0.9289	0.8764
Figure 7d	0.7603	0.6633	0.8291	0.7238

**Table 2:** CIEDE2000colordifference of the four methods.

Image	Ng	HoTVL1	Proposed Method	Multiscale Retinex
Figure 7a	26.8927	29.6729	26.6182	27.9038
Figure 7d	24.5474	26.0115	21.9972	25.4528

**Table 3:** Recovered intensity values for regions A and B of Figure 10a.

Image		Original	Ng	HoTVL1	Proposed Method	Multiscale Retinex
Checkerboard	A	120	140	85	135	109
	B	120	180	174	230	149
